# Suspected Severe Malaria in a Sudanese Patient Affected by Sickle Cell Disease Who Was Treated with Hydroxyurea

**DOI:** 10.3390/pathogens10080985

**Published:** 2021-08-04

**Authors:** Giulia Chiopris, Stefano Maccario, Tawaddud Hassan Eisa Artaiga, Abdalrhman Ibrahim Mohamed, Manuela Valenti, Susanna Esposito

**Affiliations:** 1 Pediatric Clinic, Pietro Barilla Children’s Hospital, Department of Medicine and Surgery, University of Parma, via Gramsci 14, 43126 Parma, Italy; giulia.chiopris@gmail.com; 2 Emergency NGO Onlus, 20122 Milan, Italy; stefano.maccario@emergency.it (S.M.); gina.portella@emergency.it (T.H.E.A.); info@emergency.it (A.I.M.); manuela.valenti@emergency.it (M.V.)

**Keywords:** chemoprophylaxis, hydroxyurea, malaria, *Plasmodium falciparum*, sickle cell disease

## Abstract

Sickle cell disease (SCD) is the most common genetic disease in sub-Saharan Africa. The signs and symptoms of SCD usually begin in early childhood. Characteristic features of this disorder include anaemia, repeated infections, and periodic episodes of pain. Malaria is one of the infections that can occur in patients with SCD in endemic countries. Many guidelines recommend antimalarial chemoprophylaxis in these patients, although the debate on which drug should be used is still ongoing. Hydroxyurea (HU), which is considered a safe and effective treatment for both children and adults with SCD, seems to affect the incidence and severity of malaria, although these impacts have yet to be fully demonstrated. We report a case of an eight-and-a-half-year-old Sudanese boy with SCD treated with HU admitted for suspected severe malaria who showed a recrudescence after first-line treatment. Although he had undergone splenectomy and thus belonged to a category of patients at high risk for infectious complications, he was not receiving any malaria chemoprophylaxis. This case emphasises the importance of the routine administration of malaria prophylaxis to children with SCD living in endemic areas, even when they are treated with HU, and especially if they are at high risk for infectious complications because they have undergone splenectomy. There is an urgent need for further research to evaluate the most appropriate regimen and its optimal duration.

## 1. Background

Sickle cell disease (SCD) is a chronic multi-system disease caused by an inherited haemoglobin disorder [1]. People with this disease have atypical haemoglobin molecules called haemoglobin S (HbS), which can distort red blood cells into a sickle, or crescent, shape. The signs and symptoms of SCD usually begin in early childhood. Characteristic features of this disorder include anaemia, repeated infections, and periodic episodes of pain [2]. SCD includes a number of different diseases. The most common and severe form of SCD is the homozygous state for the β^S^ mutation called sickle cell anaemia (HbSS) [2]. Other forms of SCD are compound heterozygous states with the β^S^ mutation and one of several other abnormal HBBs [2].

SCD is the most common genetic disease in sub-Saharan Africa, with 300,000 neonates born with SCD annually [3]. Malaria is one of the infections that can occur in patients with SCD in endemic countries. In fact, the guidelines in many African countries and those produced by the World Health Organization (WHO) recommend antimalarial chemoprophylaxis in these patients [4,5]. Nevertheless, the most common form of prophylaxis is limited to the use of insecticide-treated bed nets, and the debate on which drug should be recommended is still ongoing. On the other hand, hydroxyurea (HU), which is considered a safe and effective treatment for both children and adults with SCD, seems to affect the incidence and severity of malaria, although its impacts have yet to be fully demonstrated [6,7,8]. For this reason, some experts consider HU as an appropriate drug for malaria prophylaxis, without the need to add additional drugs [6,7,8]. 

We report a case of an eight-and-a-half-year-old Sudanese boy with SCD treated with HU admitted to the Emergency NGO Paediatric Clinic in Port Sudan for severe malaria who relapsed after first-line treatment. Although he had undergone splenectomy and thus belonged to a high-risk category, he was not receiving any malaria chemoprophylaxis.

## 2. Case Presentation

An eight-and-a-half-year-old male patient from Sudan presented to our outpatient department in Port Sudan with a high-grade fever (39 °C), vomiting and episodes of shivering resembling convulsions. This patient was already being followed in our clinic for SCD (he showed an homozygous state for HbS), for which he started treatment with oral HU at a dose of 15 mg/kg/day due to a history of acute chest syndrome and several painful crises with a significant time absent from school. It is of note that one year prior, this patient had undergone splenectomy in a private hospital in Khartoum, Sudan. Despite living in a malaria-endemic area, the patient did not start malaria prophylaxis. On arrival at our hospital, the child appeared in good condition; he was febrile (39 °C), with vital signs within the acceptable limits according to age and a Glasgow Coma Scale (GCS) of 15/15. Blood tests revealed a Hb level of 7.0 g/dL (which was in line with the patient’s usual Hb levels), a white blood cell (WBC) count of 11.4 × 10^3^/µL, a C-reactive protein (CRP) level of 12 mg/dL, a platelet count of 213 × 10^3^/µL, normal kidney function and mildly altered liver function (alanine aminotransferase (ALT), 59 UI/L; aspartate aminotransferase (AST), 33 UI/L). His bilirubin level was within normal limits. His urine was clear. A rapid diagnostic test for malaria (histidine-rich protein 2 (HRP2) and *Plasmodium* lactate dehydrogenase (pLDH)-based) was positive, and the blood smear confirmed the presence of *Plasmodium falciparum* with hyperparasitaemia (++++). A diagnosis of suspected severe malaria was made in this patient affected by SCD treated with HU with a history of splenectomy.

The child was admitted to our paediatric ward and started on IV artesunate (2.4 mg/kg three times per day the first day of treatment, then once daily at 2.4 mg/kg) and IV ceftriaxone (70 mg/kg/day). HU was stopped according to the protocol. The child had a fever for the first 24 h, with normal vital signs and normal GCS scores. Because of his improved general condition and apyrexia, he was started on a 3-day course of oral artemisinin-based combination therapy on the third day of hospitalisation, and subsequently, he was discharged with instructions to complete antibiotic therapy with oral amoxicillin/clavulanic acid for 7 days at home. At discharge (on day 3), his Hb was reported as stable with a value that was normal for the patient (7.0 g/dL); however, due to the risk of the worsening of his anaemia, we asked the family to attend our clinic for a follow-up and a Hb assessment in a week.

Surprisingly, two days after discharge (on day 5), the child was brought back to our clinic because of a relapse of fever (39.5 °C), episodes of vomiting and shivering. Clinically, the patient looked unwell, although he had vital signs within the normal limits for his age. The blood smear was positive for malaria (ring stage of *P. falciparum* ++, gametocytes of *P. falciparum* ++). The complete blood count showed a decreased Hb level (6.1 g/dL), increased WBC count (38.1 × 10^3^/µL), a platelet count of 401 × 10^3^/µL, an increased lactate dehydrogenase level (LDH, 1522 UI/L), a CRP level within normal values and normal kidney and liver function. Given the suspicion of a partially treated case of severe malaria, the patient was started on IV ceftriaxone 100 mg/kg/day and quinine 10 mg/kg IV three times per day. The patient completed 7 days of IV therapy. His low-grade fever disappeared completely 48 h after admission. The child underwent a blood transfusion (on day 5) because of the risk of worsening anaemia. At discharge, his Hb level was 9.1 g/dL, his WBC had normalised, and his CRP level had decreased to 6 mg/dL from the peak of 96 mg/dL (on day 8). The malaria test was repeated before discharge (on day 12), and the blood smear showed one plus for gametocytes only.

Table 1 summarises the blood examination results for this child. After one week (on day 19), the child returned for a follow-up visit: he was asymptomatic and appeared to be well, the blood tests were negative for malarial parasites, and he was able to restart his daily dose of HU. 

## 3. Discussion

Here, we report a representative case of recrudescent suspected severe malaria in a Sudanese patient affected by SCD who underwent a splenectomy at the age of 7 years. The patient was receiving regular treatment with HU and was therefore not receiving malaria prophylaxis. He developed suspected severe malaria with a recrudescence after a first-line treatment with artesunate IV, that required 7 days of treatment with IV quinine.

Subjects homozygous for HbS are at greater risk of suffering from severe malaria and have a higher mortality rate than non-SCD subjects [9]. The risks of severe anaemia and death when SCD patients develop malaria are significant [10], even though these patients are reported to have a lower parasite density [11,12]. Hyposplenism plays a significant role in the reduced response to *P. falciparum* in SCD patients, and some studies have reported a higher risk of severe malaria and a higher incidence of parasitemia after splenectomy [13,14]. Therefore, chemoprophylaxis against malaria is recommended in African guidelines for the management of SCD patients living in endemic areas [4,5]. Nevertheless, few data can be found in the literature regarding the optimal strategy of chemoprophylaxis and its safety and effectiveness in clinical practice.

Chronic therapy with HU has been shown to have clinical benefits with regard to reducing the incidences of vaso-occlusive crisis and acute chest syndrome and the need for blood transfusion in patients with SCD [6,7,8,15]. Moreover, some studies have reported an effect of HU on malaria. On the one hand, both in vitro and in animal models, HU increased the expression of endothelial intracellular adhesion molecule-1 (ICAM-1), which is a cell surface receptor responsible for the adhesion of erythrocytes infected with *P. falciparum*, facilitating parasite adhesion to the endothelium [16]. On the other hand, HU is known to increase foetal haemoglobin (HbF) levels, and some in vitro studies found a role of HbF in restricting parasite growth [17,18]. However, Mmbando et al. showed a negative epistasis between HbS and HbF and a reduction in protection against malaria [19]. Furthermore, splenic function plays an essential role in the defence against severe infections, including malaria. Studies have reported that children with SCD treated regularly with HU have preserved or even improved spleen function, ensuring a better response during infectious events [20,21]. In addition, two clinical studies explored the protective role of HU against malaria [22,23]. The first study, the NOHARM study, was a randomised double-blinded placebo-controlled trial that enrolled children with SCD in Uganda who received HU or a placebo for 12 months [22]. All participants received malaria prophylaxis with monthly doses of sulphadoxine-pyrimethamine (S-P) and anti-mosquito nets. The primary outcome was the incidence of malaria, which did not differ between the two groups, although the administration of HU was not associated with more severe malaria. On the other hand, children receiving HU had a significant increase in their Hb and HbF levels and lower incidences of painful crisis and hospitalisation. Overall, the reported malaria incidence in that study was low, reflecting the excellent adherence to oral prophylaxis and other preventive measures. No increase in the incidence of neutropenia was observed in the subjects receiving HU, confirming that HU is a safe option even in settings in which invasive infections are a cause for serious concern [22]. The second study, the REACH trial, was an open-label trial conducted in sub-Saharan Africa to assess the safety and efficacy of HU in children with SCD who were not treated with malaria chemoprophylaxis [23]. After one year of treatment, all subjects had higher levels of Hb and HbF. The incidences of painful crisis and acute chest syndrome and the need for blood transfusion were significantly reduced, as was the rate of severe infections. The authors observed a decreased rate of malaria in patients treated with HU (142.5 vs. 90 events per 100 patients/year). The benefit with regard to malaria was reported to be higher, especially after one year of treatment, with no serious adverse effects or deaths due to HU [23]. Our child with SCD, who also underwent splenectomy, developed severe malaria despite treatment with HU, indicating that malaria chemoprophylaxis is recommended for high-risk patients.

Currently, there is no consensus on the most appropriate malaria chemoprophylaxis regimen for children with SCD. The current clinical practice in most African countries includes the use of insecticide-treated nets and the early diagnosis and treatment of malaria [4,5]. Chloroquine is no longer considered an effective option for malaria chemoprophylaxis in most African countries due to the increasing resistance of *P. falciparum* [24]. A double-blind, randomised controlled trial was conducted in Senegal to evaluate the efficacy of malaria chemoprophylaxis with S-P in patients with SCD; in that study, the patients received either S-P or a placebo during the high-transmission season. The authors reported four cases of malaria, all in the placebo arm, and the patients in the S-P arm had a reduced need for blood transfusion, with no impacts on the incidences of painful crisis and hospitalisation [25]. Additionally, S-P was reported to be more effective than chloroquine at reducing the incidence of malaria in a double-blind, randomised controlled trial conducted with children with SCD in Uganda [26]. Eke et al. conducted a randomised placebo-controlled trial in Nigeria to compare proguanil, pyrimethamine and placebo in children with SCD [27]. Chemoprophylaxis was associated with a reduced need for transfusion, and proguanil appeared to be more effective at reducing parasite density than pyrimethamine and was associated with a significant reduction in the incidence of painful bone crisis [27]. A recent meta-analysis found proguanil to be the most common drug used for chemoprophylaxis in patients with SCD, showing that chemoprophylaxis is effective at reducing malaria episodes, although they did not find a significant reduction in the risk of painful crisis, the risk of hospital admission or the need for blood transfusion [28]. Considering the available evidence, proguanil, mefloquine, atovaquone/proguanil and doxycycline are the available options recommended in most African countries for chemoprophylaxis in patients with SCD, especially if they have risk factors for infections such as splenectomy [29].

## 4. Conclusions

This case emphasises the importance of administering regular malaria chemoprophylaxis to children with SCD living in endemic areas, even when they are treated with HU, and especially if they are at high risk for infectious complications because of splenectomy. There is, however, an urgent need for further research to evaluate the most appropriate regimen and its optimal duration.

## Figures and Tables

**Table 1:** Blood examination results for this child during the first and second admissions.

	First Admission			Second Admission			
	Day 1	Day 2	Day 3	Day 5	Day 7	Day 8	Day 12
Blood exams	Artesunate IV		Oral artemether/ lumefantrine	Quinine IV			
WBC, cells/µL	11,400	13,300	17,700	38,100	27,400	24,800	9500
Neutrophils	3800	7200	5300	19,800	16,300	7900	4700
Lymphocytes	6900	5000	10,700	17,000	8500	15,300	4100
Hb, g/dL	7	6.6	6.5	6.1	9	9.1	9.0
Platelet count, cells/µL	213,000	199,000	231,000	401,000	503,000	410,000	576,000
CRP, mg/dL	12		24	6	96	48	
ALT, UI/L	59			49			
AST, UI/L	33			35			
LDH, U/L	625			1522			
Bilirubin, mg/dL	1.2			1.0			
Blood smear for malaria	Positive for *P. falciparum* (++++)		Negative	Positive for *P. falciparum* (++)			Negative

AST, aspartate aminotransferase; CRP, C-reactive protein; Hb, haemoglobin; LDH, lactate dehydrogenase; WBC, white blood cell; ++++, more than 10 parasites per single oil-immersion thick film field; ++, 1–10 asexual parasites per 100 oil-immersion thick film fields.

## Data Availability

All the available data are reported in the case presentation.
